# Utility of Nephrologist-Performed Point of Care Ultrasonography in the Evaluation of Hyponatremia

**DOI:** 10.24908/pocus.v7iKidney.15339

**Published:** 2022-02-01

**Authors:** Mahmud Saqib, Gregory Capelli, Abhilash Koratala

**Affiliations:** 1 Division of Nephrology, Medical College of Wisconsin Milwaukee, Wisconsin USA; 2 Department of Medicine, Medical College of Wisconsin Milwaukee, Wisconsin USA

**Keywords:** VExUS, POCUS, venous Doppler, point of care ultrasound, hyponatremia

## Abstract

Point of care ultrasonography can be a valuable adjunct to conventional physical examination in patients with hyponatremia that aids in clinical decision making. It can address the shortcomings of traditional volume status assessment such as the inherent low sensitivity of ‘classic’ signs such as lower extremity edema. Herein, we present a case of a 35-year-old woman where discrepant clinical findings led to confusion in the accurate assessment of volume status but addition of point of care ultrasonography helped to guide the therapy.

## Case

Hyponatremia is the most common electrolyte disorder for which nephrology consultation is sought and represents an excess of water out of proportion to sodium. Accurate assessment of fluid volume status is paramount in appropriate management of hyponatremia. For example, both volume overload with decreased effective arterial volume and hypovolemia can act as stimulus for release of anti-diuretic hormone leading to hyponatremia, and the treatment is entirely different in both these scenarios. However, volume status assessment is not always straightforward owing to inherent low sensitivity of traditional physical examination signs as well as patient-related factors such as chronic lower extremity edema due to venous insufficiency 1, 2, 3. In the recent past, point of care ultrasonography (POCUS) has emerged as a valuable adjunct to physical examination in the field of nephrology 4. Rapid bedside sonographic assessment of the lung, inferior vena cava (IVC), heart, and abdominal venous Doppler waveforms in varying combinations depending on the clinical question can provide helpful insights into hemodynamics that can be integrated with conventional assessment to guide management. 

**Figure 1:**
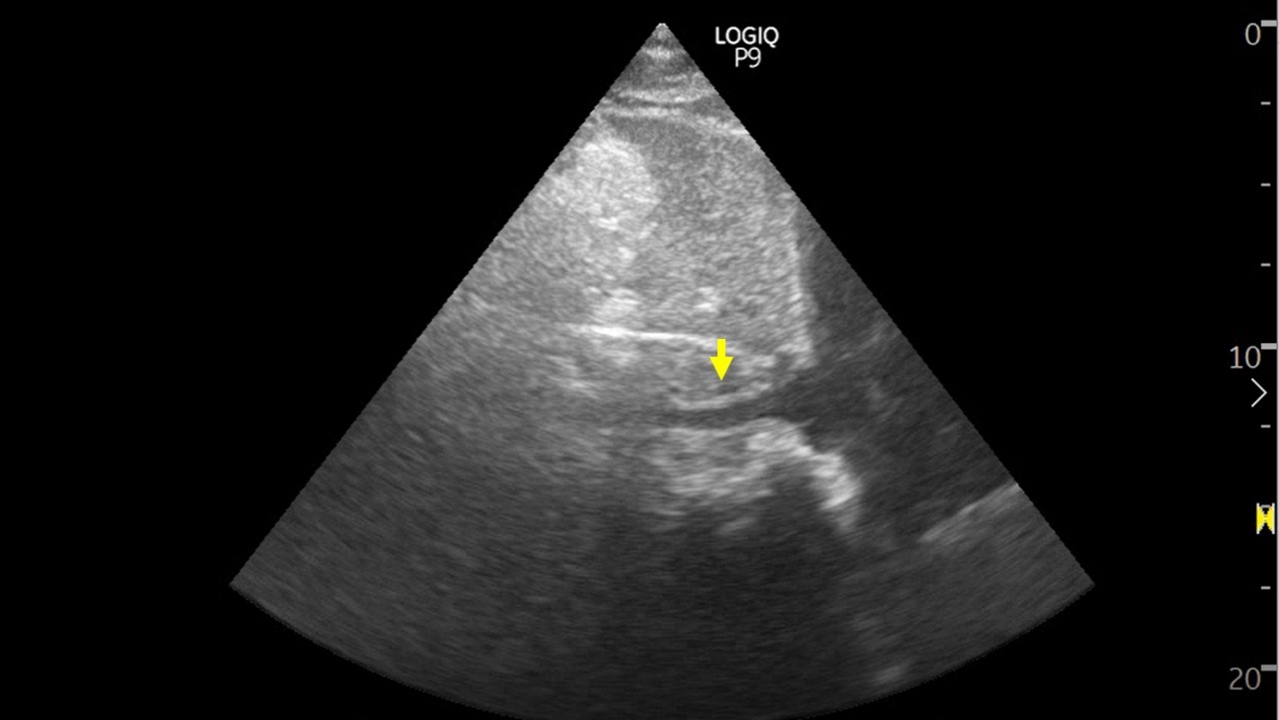
Sonographic image demonstrating a small inferior vena cava (maximal diameter). Online Video S1 demonstrates the respiratory variation. Image was obtained using a phased array transducer from the subxiphoid window with the probe orientation marker towards patient’s head.

**Figure 2:**
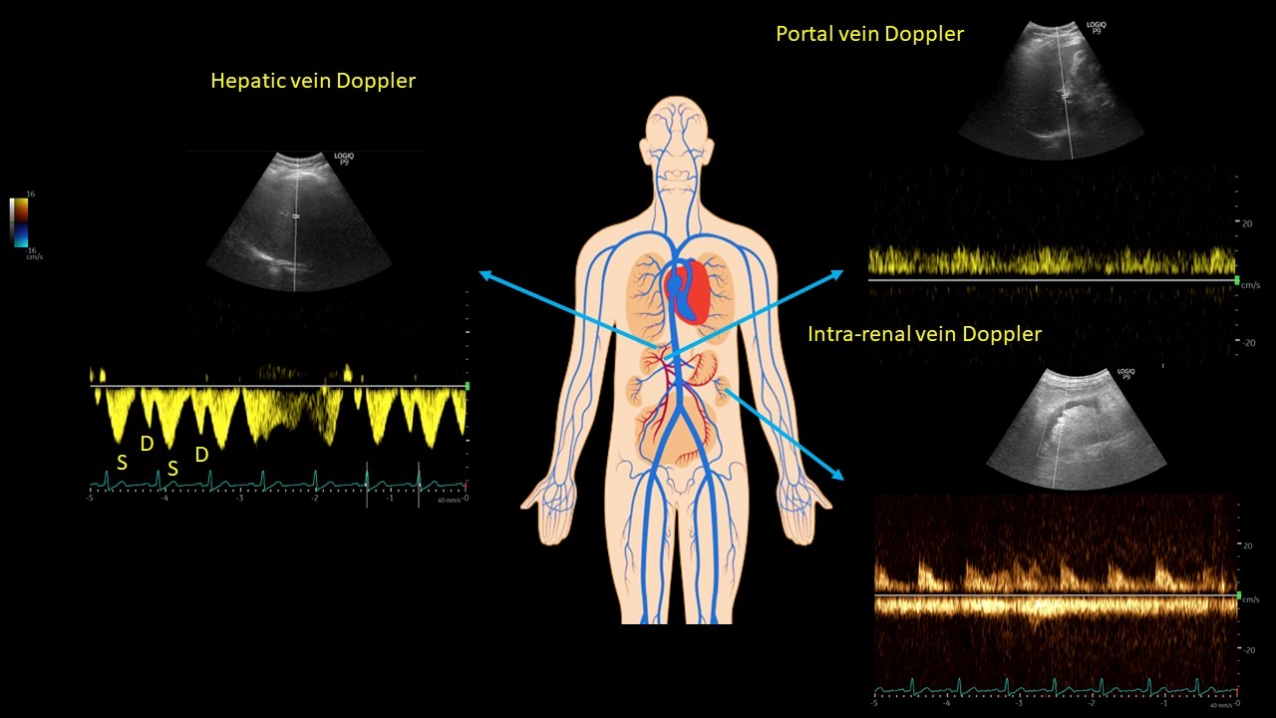
Venous Doppler images demonstrating normal hepatic, portal, and intra-renal vein waveforms. In hepatic vein, S = systolic wave, D = diastolic wave (normally, S is greater than D). For portal and intra-renal veins, a continuous pattern without distinct S and D waves is considered normal. Images were obtained using a curvilinear transducer from the lateral aspect, just below the diaphragm. Pulsed-wave Doppler was utilized to obtain the waveforms with simultaneous EKG gating. Human body image licensed from shutterstock ®

We recently cared for a 35-year-old woman with hyponatremia in whom POCUS aided in the accurate assessment of volume status. The patient had a history of developmental delay as well as a disabling connective tissue disorder with upper limb contractures and heart failure with preserved ejection fraction. She initially presented to the hospital for worsening lower extremity edema compared to her usual state. Her home dose of loop diuretic was furosemide 20 mg as needed. Laboratory data was significant for a serum sodium of 122 mEq/L (baseline: 128-130), serum osmolality 257 mOsm/kg, and a normal serum creatinine. She was treated with intravenous loop diuretics for exacerbation of heart failure and the serum sodium improved to 127 mEq/L over the next two days. Serum creatinine was relatively stable at 0.8 mg/dL. However, the sodium level subsequently declined with continued diuretic therapy and reached a value of 121 mEq/L. Nephrology was consulted for evaluation and management of hyponatremia at that time. Urine osmolality and spot urine sodium were obtained which were 259 mOsm/kg and 70 mmol/L respectively. Physical examination was significant for bilateral 2+ pitting pedal edema, but auscultated lung zones were clear. Chart review revealed a weight gain of 2 kg during the hospitalization. Based on this data, there was a discrepancy in opinion among the treating physicians about her volume status. We performed point of care ultrasound which revealed a small IVC less than 1 cm in maximal diameter with 100% collapse with inspiration suggestive of low right atrial pressure (Figure 1, online Video S1). Lung ultrasound did not reveal B-lines. Cardiac Doppler to assess fluid responsiveness could not be performed due to difficult body habitus. A venous excess Doppler ultrasound (VExUS) evaluation was performed to exclude systemic venous congestion which revealed normal waveforms in the hepatic, portal, and intra-renal veins (Figure 2). Based on these findings, hypervolemic hyponatremia was thought to be unlikely and the loop diuretics were held. In addition, fluid intake was liberalized. The serum sodium improved to 127 mEq/L over the next two days and the patient was discharged. 

Through this case, we would like to convey that POCUS can be used as a supplement to physical examination, laboratory data and other clinical parameters to evaluate volume status, and guide therapy in patients with hyponatremia. In addition, while one may question the utility of VExUS scan when the IVC is small 5, we do it in two situations. First, we use VExUS in patients with limited acoustic windows, where IVC ultrasound is prone to errors such as mistaking aorta or caudate lobe of the liver for IVC. Second, we use VExUS in those with discrepant clinical data such as our patient who had a discrepancy in weight change as well as lower extremity edema which was felt due to factors other than volume overload. 

## Conflict of Interest

The authors have declared that no conflict of interest exists.

## Patient consent

Informed consent was obtained from the patient’s guardian to publish this case study. No identifiable information included.

## Authorship

All the authors have made substantial contribution to the preparation of this manuscript. 

## Supplementary Material

 Video S1 Sonographic image demonstrating a small inferior vena cava (maximal diameter). This video demonstrates the respiratory variation. Image was obtained using a phased array transducer from the subxiphoid window with the probe orientation marker towards patient’s head.
